# Crystallising places: Towards geographies of ontogenesis and individuation

**DOI:** 10.1177/03091325231224042

**Published:** 2023-12-23

**Authors:** Peter Merriman

**Affiliations:** 1 Geography and Earth Sciences, 1026 Aberystwyth University, Aberystwyth, UK

**Keywords:** Place, difference, assemblage, Deleuze, Simondon, process, becoming, individuation

## Abstract

Building upon the current concern with relational, processual and assemblage approaches to place, this paper argues for a move away from ‘pointillist’ and constructivist accounts of the assembling of places because they reinforce binaries, reintroduce structures and highlight singular representational moments in the building, identification and dismantling of places. Drawing upon Deleuze’s philosophy of difference and Simondon’s writings on individuation, I suggest that places can more usefully be seen as events of crystallisation, distillation or folding characterised by multi-phased processes of individuation through which distinctive ‘individual-milieu coupling[s]’ emerge, refocusing attention on the open-ended, plural and eventful qualities of places-in-becoming.

## I Introduction

…one must be incredulous about the polarization of place and space, which hinges on the glaciation of events in perpetual process. If it were not such an inelegant neologism, I would be tempted to say that there is nothing but *splace*, taking splace – *splacing*. (Doel, 1999: 9)

Space and place have long served as important keywords in anglophone human geography and cognate spatial disciplines, being approached through a broad array of epistemological positions, sub-disciplinary lenses, theoretical approaches and research methodologies (see, for example, Cresswell, 2015, 2019; Merriman, 2022). Space and place have been approached as verbs and nouns, static and dynamic, material and ethereal, measurable and locatable, open and closed, produced and performed, a priori and *a posteriori*, and as cultural, political and economic productions. When opposed or held in tension, many observers have echoed Yi-Fu Tuan’s sentiments, asserting that ‘“space” is more abstract than “place”’, and that ‘what begins as undifferentiated space becomes place as we get to know it better and endow it with value’ (Tuan, 1977: 6). In contrast, other thinkers – often drawing upon phenomenological and anthropological thinking – have maintained the distinction, but effectively reversed Tuan’s ontological positioning of space as prior to place, arguing that space is a peculiarly Western abstraction, and that we are all immersed and dwell in highly variegated, particular and locally embedded places (e.g. Certeau, 1984; Ingold, 2000, 2009). As Edward Casey once remarked in an essay on anthropology, phenomenology and place:

What if the very idea of space is posterior to that of place, perhaps even derived from it? What if local knowledge… precedes knowledge of space? Could place be general and “space” particular? (Casey, 1996: 16–17)

Referencing earlier work in Gestalt psychology, the eminent anthropologist Marilyn Strathern referred to Casey’s switching around of place and space as a ‘figure-ground reversal’ (2002: 88). This assertion of dynamic, mobile and immersive practices of placing and place-making as somewhat generic and universal processes, and the positioning of ‘places’ as quasi-material, shifting contextual grounds or frames, echoes the emphasis of other scholars on the processual and more-than-representational aspects of spatial relations, practices and events. In the most committed iterations of poststructuralist and processual theories, thinkers have refused – as Marcus Doel does, above – to distinguish between space and place, while also denying the settlement, identification, bounding, closure and representation of places or spaces as points, areas or things. Points and ‘pointillism’ (Doel, 1999) are rejected because they reproduce an appearance of stasis, independence and singularity, whereas places are always plural, open to elsewheres, eventful, becoming, in process and practised. Our focus, then, must be on spacing and/or placing:

*To space* – that’s all… There is just spacing (differentials). The “points” – as things, events, terms, positions, relata, etcetera – that are supposedly played out “upon” and “alongside” space are illusory. Space is immanent. It has only itself. (Doel, 2000: 125)

Between the two extremes – of scholars who treat space and place as singular, material, dimensioned, and/or measurable formations *and* those who conceive spacing and placing as ‘pointless’, multiple, processual events – one can identify a whole host of thinkers who seek to advance anti-essentialist, non-binary, processually sensitive, dialectical and/or politically attuned approaches to the entangled production, structuring and individuation of spaces, places and subjects.

In this paper, I provide a critical discussion of different processual approaches to place and placing, paying particular attention to scholars who draw upon the writings of Gilles Deleuze and Félix Guattari to approach place through the concepts of becoming, assemblage and re/de-territorialisation. While Marcus Doel advocates ‘pointless’ approaches which emphasise the becoming, placing and spacing of events, I show how geographers frequently integrate Deleuzoguattarian thinking into constructivist and representational frameworks in partial (and, arguably, compromised) ways which reinforce binaries, reintroduce structures and highlight singular moments in the assembling, building, identification and dismantling of places. While this may provide a practical way to incorporate poststructuralist thinking into social scientific studies committed to a ‘know-and-tell’ methodology and epistemology (Dewsbury, 2010: 321), I would suggest that there are other ways of approaching place which are more experimental, open-ended and propositional in their style of theorising and practising, while also remaining committed to plural and multi-phased accounts of the becoming of events and subjects.

In section two, I trace the emergence of processual approaches to place at various points in the history of human geography, before proceeding, in section three, to focus on geographers who have drawn upon the writings of Deleuze and Guattari to approach place through concepts like territory, the fold, topology, milieu, refrain, deterritorialization, reterritorialization and assemblage. I argue that while these approaches can be used to focus on the incessant becoming of places, the tendency of geographers to rely upon binary understandings of reterritorialization/deterritorialisation, assembly/disassembly and absence/presence enrols such theoretical approaches into representational and constructivist accounts which emphasise the building and settling of worlds and de-emphasise or background incessant transversal processes of folding, distillation and deformation. I then argue that Deleuze’s philosophy of difference-in-itself can hold invaluable lessons for geographers seeking non-representational and non-identarian approaches to place, before proceeding, in section four, to discuss recently translated writings by the French philosopher Gilbert Simondon on individuation. Simondon draws upon theories from crystallography, thermodynamics, psychology, sociology and philosophy to highlight the multi-phased processes of physical, social and collective individuation through which beings, material entities and environmental milieu emerge and become differentiated. I argue that Simondon’s trans-disciplinary theoretical approach is well-suited to the heterogeneous properties of places and environments which emerge as individuated distillations or crystallisations characterised by distinctive multi-phased ‘individual-milieu coupling [s]’ (Simondon, 2020 [2005]: 3). In doing this, I aim to show how processual understandings of the individuation and crystallisation of places can help to refocus the attention of geographers on the open-ended, dynamic, plural and eventful qualities of places.

## II Place and process in human geography

The river where you set your foot just now is gone – those waters giving way to this, now this. (Heraclitus, 2003 [c.500 BCE]: 27)

Processual thinking has a long and complex history, foregrounding movement, flux and becoming, and challenging the tendency of many thinkers to reduce dynamic processes and events to particular points in space and time. Prominent ‘Western’ thinkers – from Ancient Greek philosophers such as Heraclitus and Lucretius, to Henri Bergson, Alfred North Whitehead and, more recently, Michel Serres, Gilles Deleuze, Jane Bennett and Erin Manning – have questioned the ways in which process, movement, change, flux, turbulence, vibration and unfolding are commonly approached as subsidiary to primary or foundational concepts such as space, time, matter, energy and force (Merriman, 2012b; Nail, 2019). Within geography, forward-thinking scholars such as Percy Crowe (1938), Derwent Whittlesey (1945) and Jim Blaut (1961) argued for an attention to space, time *and* movement, while the emergence of humanistic approaches in the 1970s – and the advance of more critical conceptualisations of place – led a few prominent geographers to approach place in more dynamic, processual and relational terms. Anne Buttimer (1976: 282) advanced a phenomenological geography in which people’s dynamic lifeworlds were seen as ‘in process of becoming’, while David Seamon drew upon phenomenology, existentialism, behavioural geography and time geography to conceive places as dynamic, organic, rhythmic, synergistic environments which may usefully be conceived as ‘place-ballets’ fusing ‘many time-space routines and body-ballets in terms of place’ (Seamon, 1980: 159). Buttimer and Seamon’s accounts appeared in stark contrast to Yi-Fu Tuan’s conclusions that as ‘an organized world of meaning’, place ‘is essentially a static concept’ (Tuan, 1977: 179).

Humanistic geographers were famously criticised by feminists for presenting human experience as rather singular, unpositioned and universal, reinforcing masculinist constructions of space and place, while Marxists highlighted a neglect of broader issues of power, inequality, and social and political processes and structures (Rose, 1993). A number of time geographers and structuration theorists attempted to develop more synthetic approaches spanning different traditions, with Allan Pred combining elements of structuration theory, time geography, humanistic geography and the work of Raymond Williams to focus ‘on the becoming of sense of place’ (Pred, 1983: 45) and ‘place as historically contingent process that emphasizes institutional and individual practices as well as the structural features with which these practices are interwoven’ (Pred, 1984: 280). Place is figured as: becoming; not static; ‘taking place’ (Pred, 1983: 53); or ‘unfolding’ (Pred, 1984: 282; 1986: 195). The language Pred utilised to capture the dynamic unfolding of places is striking for its resonance with more recent processual and non-representational thinking, and this should come as no surprise, for Pred was a friend and key ally of Nigel Thrift, who started his career as a time geographer in the 1970s, critically engaging with structuration theories in the 1980s, and developing poststructuralist, non-representational theories of practice in the 1990s (see Thrift, 1983, 1996).

Critical political and particularly Marxist geographers have also engaged with processual understandings of place. Writing in *Justice, Nature and the Geography of Difference*, David Harvey (1996) drew upon a vast array of thinking – including the writings of Leibniz and Whitehead, as well as Marx – to advance a dialectical and processual approach that saw ‘the process of place formation’ as one of ‘carving out “permanences” from the flow of processes creating spatio-temporality’ (Harvey, 1996: 261). Similarly, in *The Nature of Space*, the Brazilian geographer Milton Santos drew upon processual thinking – including Whitehead, Simondon and Serres – to assert that ‘places… can be seen as intermediaries between the world and the individual’, forming ‘a tense reality, a dynamism recreated in each moment’ (Santos, 2021 [1996]: 215–216). More influential in anglophone geography, have been the writings of Doreen Massey, who adopted relational, pluralistic and processual approaches to space and place in much of her later work, while also refusing to define place and space (and space and time) through their opposition (Massey, 1991, 1992). In the early 1990s, Massey insisted on considering space and time together as ‘four-dimensional’ space-time (Massey, 1992), while also advancing a left-leaning progressive ‘global sense of place’ in which ‘local’ places are approached as ‘extroverted’ meeting places in which global spatial processes become grounded (Massey, 1991: 28). Massey was keen to dismantle common binaries which counter-posed open, dynamic, generic, progressive and/or globalised *spaces* with closed, static, reactionary and local *places*; integrating the two concepts in a progressive relational framework which positioned spaces and places as neither static nor as ‘all flow’. In her book *For Space*, Massey drew upon the poststructuralist theories of Derrida, Deleuze and Guattari alongside works from political theory to develop a politics of space-times and places conceived as open, woven, temporary, ‘throwntogether’ constellations in process and becoming (Massey, 2005: 21, 131, 140–141), critiquing the counter-posing of space and place by phenomenological and existential thinkers such as Martin Heidegger, Ed. Casey and Yi-Fu Tuan (*ibid*.: 183).

What this broad spectrum of work reveals is how geographers have long recognised the importance of emphasising the becoming, processual formation or unfolding of places. Over the past three decades, this has not simply been advanced through traditions of process philosophy, but also theories of dialectics (Harvey, 1996), actor network theories (Murdoch, 1998; Bingham and Thrift, 2000), rhythmanalysis (Lefebvre, 2004 [1992]; Mels, 2004; Edensor, 2010), post-phenomenology and post-humanism (McCormack, 2017; Ash, 2020), and an array of empirically grounded writings in cognate disciplines like anthropology (e.g. Ingold, 2009; Dovey, 2010). Despite these various attempts to develop more dynamic, open and processual understandings of space and place, I would argue that much of this work still ‘bears the trace[s] of pointillism’ (Doel, 1999: 9), holding on to constructivist and representational logics which explain the building of worlds by human and non-human actors, while failing to account for the ontogenetic processes through which places are individuated, deformed, folded, crystallised and vibrate. While this residual pointillism may ground geographic discourses in commonsense spatial vocabularies which speak across disciplinary boundaries and reach into policy spheres, it may also unwittingly reinforce very particular Western ontological assumptions and frameworks and lack the theoretical experimentation that could provide an all-important ‘shock to thought’ (Massumi, 2002).

## III Place and process after Deleuze (and Guattari)

Over the past three decades a number of influential spatial thinkers have approached spatial questions through the joint and solo writings of Gilles Deleuze and Félix Guattari. While a good deal of attention has focussed on concepts such as smooth and striated space, rhizomic and arborescent spatial networks, geophilosophy, and spaces of affect, a growing body of writing has worked through the implications of Deleuze and Guattari’s processual philosophies of becoming for how scholars conceive place and places (e.g. Doel, 1996, 1999, 2000; Buchanan and Lambert, 2005; Conley, 2012; Saldanha, 2017).

### 1 Folds, territories, assemblages

A first important concept in this work is the action of (un)folding. Writing in 1996, Marcus Doel outlined Deleuze and Guattari’s processual and ‘scrumpled geography’, arguing that ‘the individuation of a molar aggregate – such as a thing, person, place, concept, or animal – takes on consistency, turgescence, and rigidity by way of a regulated practice of folding’ (Doel, 1996: 436). Deleuze’s later solo writings on Foucault and Leibniz are mobilised to shatter ‘pointillist’ geographies of place-delineation, place-making, and individual-milieu relations that approach place ‘in terms of character, meaning, awareness or consciousness’ (Doel, 1999: 8). Pointillist approaches consider places to be individualised molar aggregations, thus obscuring the micropolitical and molecular practices of folding which cut across distinctions between interior/exterior, subject/object and process/product (Doel, 1996, 1999). In Doel’s (1999: 10) poststructuralist approach, ‘Geography is simply an inclination towards the event of spacing’, and he joins others in looking to non-Euclidean geometric traditions like topology to capture the complex, non-linear relations and processes involved in the unfolding of events (as spacing and placing), something that is evident in Deleuze and Guattari’s own references to Riemann space, Lucretian physics, Mandelbrot’s fractals and the Sierpinski sponge (Deleuze and Guattari, 1988 [1980]: 482–490). Kevin Hetherington’s (1997: 197) folded ‘topological arrangements’ are clearly similar to Doel’s ‘scrumpled geography’, as are the ‘Baroque geographies’ discussed by Deborah Dixon and John-Paul Jones (2014), and these approaches to places folding or becoming folded were echoed in the non-representational spatialities traced out by thinkers such as Nigel Thrift and John Wylie, for whom Deleuze’s writings on the fold could usefully be considered alongside Maurice Merleau-Ponty’s post-phenomenology of the twisting and folding of the ‘flesh’ (*la chair)* of the world (Thrift, 1996; Wylie, 2006). Whereas Doel (1999) rejects all spatial pointillism, Thrift adopts a ‘radically contextual’ *regional* approach to time-spaces and places, which assumes that ‘the subject can only “know from”’ a particular time-space context which is ‘a performative social situation, a plural event which is more or less spatially extensive and more or less temporally specific’ (Thrift, 1996: 41, 39, 41). In Thrift’s writings, space and place seem to retain a minimal pointillism (Doel, 1999: 10), although this is less pronounced in later writings where he approaches them through concepts of practice, becoming, movement, performance, event, turbulence, folding and a language emphasising ‘distribution, dynamism, effort and friction’ (Thrift, 1999: 308).

A second concept which is important for Deleuzoguattarian conceptions of place is territory, around which they also develop the concepts of deterritorialization, reterritorialization, milieu and refrain. Writing in *A Thousand Plateaus*, Deleuze and Guattari outline the continual processual movements of deterritorialization and reterritorialization by which territories are simultaneously formed, dismantled and always in a state of becoming. Territories are ‘not primary’ but are ‘products of territorialization’, emerging from expressive acts, rhythms and milieus which ‘cease to be directional… [and] functional’, ‘becoming dimensional,… expressive’, and territorialised (Deleuze and Guattari, 1988 [1980]: 315). Arun Saldanha (2017: 106) suggests that ‘territory’ is Deleuze and Guattari’s ‘generic term for what geographers and others have called place’, but there is nothing generic about the way they develop the concept of territory alongside the twin processes of deterritorialization and reterritorialization, which are exemplified through a series of specific discussions of animal and bird life, capitalism, state politics and music. Key to this outlining of reterritorialization and deterritorialization are the concepts of refrain, milieu and assemblage. A refrain or *ritornello* is a territorialised ‘rhythm and melody’ in a process of becoming ‘expressive’ (Deleuze and Guattari, 1988 [1980]: 317). Refrains may be sonorous, gestural and motor – as in the example of a fearful child, humming or singing while walking in the dark (Deleuze and Parnet, 2006 [1977]: 73) – and their passage or movement may act to reterritorialize or deterritorialize new and existing assemblages:

‘The refrain moves in the direction of the territorial assemblage and lodges itself there or leaves. In a general sense, *we call a refrain any aggregate of matters of expression that draws a territory and develops into territorial motifs and landscapes* (there are optical, gestural, motor, etc., refrains)’ (Deleuze and Guattari, 1988 [1980]: 323).

The concept of *milieu* is also integral to Deleuze and Guattari’s discussion of territory, de/re-territorialisation and the refrain, reflecting the multiple meanings of *milieu* in French as ‘surroundings’, ‘medium’ and ‘middle’ (Massumi, 1988: xvii). Milieus are seen to emerge from chaos, acting as ‘vibratory… block[s] of space-time constituted by the periodic repetition of the component’ (Deleuze and Guattari, 1988 [1980]: 313). Milieus undergo a continual coding, decoding, movement, deterritorialization and reterritorialization, and this results in milieus becoming consolidated as ‘territorial assemblages’ (Deleuze and Guattari, 1988 [1980]: 329).

It is this final concept – assemblage – which has probably received the most attention by geographers over the past decade, although a number of scholars have focussed on the problem of translating the French term *agencement* to ‘assemblage’, which does not really articulate its processual meanings as a verb as well as a noun – as in a process of arrangement (Phillips, 2006; McFarlane and Anderson, 2011; Buchanan, 2015, 2021; Manning, 2016).^1^ Assemblage theories have proved popular amongst scholars seeking to combine elements of relational and poststructuralist thinking with empirical research to form distinctive realist approaches to socio-spatial formations or places. The result, as George Marcus and Erkan Saka point out, is that assemblage serves as something of a ‘structure-like surrogate’, ‘an anti-structural concept that permits the researcher to speak of emergence, heterogeneity, the decentred and the ephemeral in nonetheless ordered social life’ (Marcus and Saka, 2006: 101).^2^ In their important review of assemblage theories and geography, Ben Anderson et al. argue that this thinking ‘offers four things to social-spatial theory’:

an experimental realism orientated to processes of composition; a theorization of a world of relations *and* that which exceeds a present set of relations; a rethinking of agency in distributed terms and causality in non-linear, immanent, terms; and an orientation to the expressive capacity of assembled orders as they are stabilized and change. (Anderson et al., 2012: 171).

Central to this work have been the writings of philosopher Manuel DeLanda – whose ‘assemblage thinking’ shows important departures from Deleuze and Guattari’s thought (see DeLanda 2006, 2016) – and its interpretation by the planning theorist Kim Dovey (see Dovey 2010, 2020). Dovey, in particular, has quite clearly argued that places should be approached as assemblages, and this work has informed the writings of a number of geographers on place (e.g. J. Anderson, 2012; Cresswell, 2019; Woods et al., 2021).

### 2 Assemblage thinking and place

In a paper on ‘the surfed wave as assemblage and convergence’, Jon Anderson (2012) draws upon the writings of Deleuze and Guattari, DeLanda, Dovey, Ingold and others to argue that places are constituted by both ‘things’ and ‘practices’, disassembling and reassembling while in a continual state of becoming. Assemblage thinking is used to underpin the idea that ‘surfers, boarders, and waves are “connected” together to form one coherent unit for the lifetime of the ride’ (*ibid*.: 570), but this constitutive or constructive ‘“brick” by “brick”’ approach is also seen to have distinct limitations, and Anderson draws upon the concept of convergence to try and incorporate some of the processual connotations of the French term *agencement* (*ibid*.: 582). In *Maxwell Street: Writing and Thinking Place,* Tim Cresswell (2019) draws upon assemblage thinking in advancing a meso-theoretical realist approach to place ‘as a gathering of things, meanings, and practices’ (*ibid*.: 169). Drawing upon DeLanda’s assemblage thinking, he explores how places are territorialised and deterritorialised, gathering and dispersing, in process, and ‘becoming and dissolving on a daily basis’ (*ibid*.: 175). Similarly, in a paper on ‘Assemblage, place and globalisation’, Woods et al. draw upon assemblage thinking to understand the relation between global processes and local places, drawing upon detailed empirical research to understand how a series of named places in different parts of the world have become assembled and dismantled through processes of coding and ‘cycles of territorialisation, deterritorialisation, and reterritorialisation’ (Woods et al., 2021: 288).

While all of these writings on assemblage and place discuss the processual aspects of places as becoming, and demonstrate a clear awareness of the constraints of the English term ‘assemblage’ (as opposed to the French *agencement*), they are also all clearly committed to advancing realist approaches to the construction, building and disassembling of places and therefore remain entrenched in the sedentarist, pointillist and singular ontologies which underpin everyday life in Western societies. While each, in their own way, acknowledges the limitations of bounded and pointillist conceptions of place – foregrounding issues of becoming – none of them suggests an approach to place which is not grounded in the kinds of pointillist and conjunctive ‘building block’ geographies identified by Doel (1999). At root, then, assemblage thinking appears to be too entangled with the everyday calculative logics of addition/subtraction and multiplication/division. These are ultimately grounded in Euclidean-Cartesian architectonic spatial logics for understanding the assembling/disassembling, construction/deconstruction, and building/demolition of dimensioned places and worlds, brick-by-brick, even where subjective apprehensions of these worlds are considered, and the ‘number’ of worlds multiplied as a result. The problem, then, is not simply that assemblage thinking focuses upon products or things above processes and events, but that it fails to account for the incessant becoming of places through events which can more usefully be understood as emerging through transversal and non-linear folding actions. This is a world which Marcus Doel (1999) approaches through the *practices* of origami, not the end products of origami or the practices and products of ‘brick-laying’. It is these kinds of alternative figurings which social science and humanities scholars have tried to articulate in theoretical discussions of ‘fire-space[s]’ (Mol and Law, 1994; Law and Mol, 2001), ‘topology’ (Hetherington, 1997; Martin and Secor, 2014), ‘phase spaces’ (Jones, 2009; Ash, 2018) or ‘movement-spaces’ (Thrift, 2004; Merriman, 2012a, 2012b), emphasising the contingency, dynamism, processuality, plurality, non-linearity and irreversibility of processes of ‘placing’ and ‘spacing’ – where this ‘plurality’ involves the imbrication and unfolding of an innumerable number of sentient subjects, subject positions and events of apprehension, all at once.

### 3 Difference-in-itself and non-representational approaches to place

This focus on the becoming of places, inspired by Deleuze and Guattari’s thinking in *A Thousand Plateaus*, is important, but this line of thinking is evident throughout many of Deleuze’s earlier texts outlining his philosophy of ‘difference-in-itself’ which would go on to inform the earliest work on non-representational theory in geography. Indeed, it has often been remarked that *Difference and Repetition* is ‘the keystone for Deleuze’s work as a whole’ (Williams, 2013: 2), especially his broader project of tracing a non-representational and processual philosophy based upon the thinking of Baruch Spinoza, Gottfried Leibniz, Henri Bergson, Gabriel Tarde, Gilbert Simondon and others. It is for this reason that Dan Cockayne et al. (2017) argue that geographers *should* pay more attention to *Difference and Repetition*, as it can provide important insights into Deleuze’s influential later work, which has strongly influenced geographic thinking over the past two decades. Nevertheless, I would argue that it is only in Doel’s writings that the implications of Deleuze’s thought for thinking space and place are pursued to the full. Thus, the long-standing interest of geographers in the processual and dynamic aspects of places and place-making, and the focus of many scholars on the cultural, economic and political dimensions of place identification, bounding and representation, reveals a reliance upon concepts of ‘difference’ which are founded on opposition and negation, and ‘remain… subordinated to identity’ (Deleuze, 2004 [1968]: 61). There are important exceptions, but conventional, ‘common-sense’ approaches to ‘difference’ all-too-frequently present it as an *a posteriori*, secondary result of comparisons between pre-constructed, discrete pointillist ‘things’, and these have come to underpin a broad array of approaches in Western philosophy and the social sciences. Difference is, here, taken to both reflect and reinforce representational approaches to identity, subjectivity and objectivity, and Deleuze’s response in *Difference and Repetition* (2004 [1968]) is to assert that ‘identities are only simulated, produced as an optical “effect” by the more profound game of difference and repetition’ (Deleuze, 2004 [1968]: xvii). The alternative approach is for scholars to refocus their attention from differences between *things* to differential *processes*, thinking ‘difference-in-itself independently of the forms of representation which reduce it to the Same, and the relation of different to different independently of those forms which make them pass through the negative’ (Deleuze, 2004 [1968]: xvii-xviii).

Deleuze’s rethinking of difference and repetition through an attunement to ‘internal difference’, tendencies, duration and the virtual is evident in his early essays on Henri Bergson from the 1950s and 1960s (Deleuze, 2004 [1956]; 1991 [1966]). For Bergson, Deleuze claims, ‘our condition condemns us to live among badly analyzed composites, and to be badly analyzed composites ourselves’ (Deleuze, 1991 [1966]: 28), and we could echo this Bergsonian-Deleuzian critique to insist that *places* frequently appear and are conceived in both everyday life and scholarly debate as ‘badly analysed composites’ (see Thrift, 1999: 303). This is what Marcus Doel is referring to when he argues that geographers remain ‘hung up on points: sites, places, nodes’ (Doel, 2000: 120) and need to focus their attention on difference-in-itself and the event of placing rather than pointillist representations and identities of ‘places’:

The world that takes place is not simply the addition of reality to a prefigured possibility… The world that returns is never the same world. What returns with the taking place of the world is neither the same, nor the identical, nor the possible – but the event. (Doel, 2010: 121)

This desire to understand the formation and transformation of composites, representations or identities underpins many engagements with theories of assemblage/*agencement*, but the frequent figuration of assemblages through a pointillist logic of addition/subtraction does not sit well with Deleuze’s philosophy of ‘pure difference’ and difference-in-itself, which is conceived as non-dualistic, non-representational, mobile and continuous:

the primary difference, which concerns those differences that carry their reason within themselves, is not the one staged between differences of degree and differences in kind, but rather resides in the differences that belong to intensity and which inform both, so constituting the real nature of difference (differences constituted by temperature, pressure, tension, potential, etc.). Where the difference of degree denotes the extensity that ex-plicates difference, the difference of kind names only the “quality” that is im-plicated in this extensity. What is left out of the picture for Deleuze is that which lies beneath the two orders, namely, the “intensive”. (Ansell Pearson, 1999: 74)

Intensity emerges as a key concept in *Difference and Repetition* (2004 [1968]), and we could indeed conclude that ‘Difference *is* intensity’ for Deleuze (Ansell Pearson, 1999: 74). ‘Intensity’ and ‘intensive properties’ are key to understanding the relationship between *virtual* potentials and *actual* extended objects (Clisby, 2015), where Deleuze focuses on the ongoing practices or tendencies entailed in the ‘differenciation’ [*différencier*] by which there is a ‘movement of the virtual towards its actualization’ (Boundas, 1996: 91), and the processes of ‘differentiation’ [*différentier*] by which ‘the virtual content of an Idea as problem’ is determined (Deleuze, 2004 [1968]: 261). More significantly for us, though, the concept of intensity is central to Deleuze’s reading of Simondon’s philosophy of individuation, for ‘intensity provides the impetus for individuation to occur’, enacting the process through which the *virtual* ‘pre-individual field’ conceived by Simondon is *actualised* into extensive ‘specific cases of individuation’ (Clisby, 2015: 141).

Deleuze’s concepts of difference and repetition, and his couplets of the virtual and actual, differenciation and differentiation, intensity and extensity, and singularity and multiplicity, emerge from his engagement with a broad range of traditions of thinking in philosophy, biology and mathematics. When reading *Difference and Repetition* it is easy to get caught up in the formalities and functional relationships presented through Deleuze’s technical language, and this is where Simondon’s writings on ‘individuation’ and ‘associated milieu’ can be useful for clarifying Deleuze’s non-representational approach, and for rethinking place and the processes of place-making. Simondon’s writings on *Individuation*, I argue, can enable scholars to refocus their attention from the processes of *agencement* (assemblage) involved in the *construction* of individual places to the *individuation* and *ontogenesis* of places and bodies from their associated milieux.

## IV Gilbert Simondon, ontogenesis and the individuation of places

Individuation does not presuppose any differenciation; it gives rise to it. Qualities and extensities, forms and matters, species and parts are not primary; they are imprisoned in individuals as though in a crystal. (Deleuze, 2004 [1968]: 308–309)

Deleuze’s philosophy of difference points towards processual philosophies of ontogenesis, individuation and becoming rather than rigid accounts of fixed, closed individual beings. His arguments in *Difference and Repetition* draw heavily upon the work of Spinoza and Simondon, as well as Duns Scotus, Raymond Ruyer and Gabriel Tarde, and the consequences of his thinking on individuation, difference, identity, representation and relationality can be seen across many other works – from ‘his definition of the individual as a multiplicity’ in *Logic of Sense* (2015  [1969]), to criticisms of the ‘hylomorphic model’ of rigid ‘form-matter’ in *A Thousand Plateaus*, and his more Leibnizian take on individuation in *The Fold* (2006  [1988]) (Williams, 2013: 231). As a result, social theorists – including geographers – have been paying increasing attention to thinkers who have informed Deleuze’s writings on difference, and this has included a renewed attention to philosopher Gilbert Simondon’s thinking on individuation, ontogenesis, information and technics. My assertion, here, is that Simondon’s thinking – particularly his recently translated writings on *Individuation* – can provide a useful lens for rethinking geographical approaches to place and placing.

Gilbert Simondon (1924–1989) is best known in both francophone and anglophone scholarship for his theoretical approach to technical objects and human/machine relations, as outlined in his secondary or adjunct doctoral thesis and first book, *Du Mode d’Existence des Objets Techniques *(see 2017  [1958]). Despite a full English translation only becoming available in 2017, the book has been widely discussed in English language social theory, media studies and cultural geography for several decades.^3^ This is in part because he provides a forward-thinking, relational approach to technical being which considers the ontogenetic relations between elements, individuals and ensembles, ‘articulates technological reality in *ecological* terms’, and frames life as a kind of being or becoming with technical objects and machines (Lindberg, 2019: 299). In contrast, much less attention has focussed on Simondon’s principal doctoral thesis of 1958, the first part of which was published in book form in 1964, with the second part only appearing in 1989 (Scott, 2014). With the unification of both volumes in English as *Individuation in Light of Notions of Form and Information* in 2020, anglophone scholars are now able to explore Simondon’s highly original and distinctive philosophy of individuation which heavily influenced Deleuze’s thinking, while also bridging the gap ‘between his thinking on technics and his philosophy of individuation’ (LaMarre, 2013: xiv). In the introduction to *Individuation*, Simondon opens with a critique of two prevailing approaches to individual being: first, the ‘self-centred monism of substantialistic thought’ and atomist philosophy which ‘considers the being as consisting in its unity, given to itself, founded on itself’; and second, a ‘hylomorphic schema’ through which individuals are seen to emerge ‘by the encounter of a form and a matter’ (Simondon, 2020 [2005]: 1). Both approaches position the individual as having a clearly represented and representable, stabilised and at times ‘final’ form and identity, but rather than see ‘individuation’ as a momentary process resulting in forms, individuals and ontologies, Simondon’s radical move is to focus on ongoing processes, phases, operations or events of a ‘being’s becoming’ (*ibid*.: 4). This requires an attention to processes of ‘information’, ‘individuation’ and ‘ontogenesis’ (*ibid*.: 3, 53):

…we would try to grasp ontogenesis in the whole unfolding of its reality and *to know the individual through individuation rather than individuation starting from the individual*. …The individual would then be grasped as a relative reality, a certain phase of being which supposes a pre-individual reality prior to it and which, even after individuation, does not fully exist all by itself, for individuation does not exhaust in a single stroke the potentials of pre-individual reality, and, moreover, what individuation manifests is not merely the individual but the individual-milieu coupling. (*ibid*.: 3)

Simondon’s understanding of ‘pre-individual being’ is grounded in his interpretation of field theories, quantum theories and metastable crystalline solutions (Simondon, 2020 [2005]: 6; Garelli, 2020; Sauvagnargues, 2013). ‘Pre-individual being’ occurs ‘before every phase’ of individuation, and it is anterior but not temporally prior to individual being (Simondon, 2020 [2005]: 361). ‘Pure pre-individual life’ can thus be seen as one of Simondon’s three ‘vital systems’ – alongside ‘meta-individual forms’ and ‘totally individualized forms’ (*ibid*.: 188) – revealing a charge or potential for individuation, but possessing ‘no individual or collective contents’ (Grosz, 2017: 172).

It would be an error to assume that processes of ontogenesis and individuation involve monadic human subjects or groupings of quasi-monadic subjects. Simondon (2020a [2005]) posits ontogenesis and individuation as primordially environmental or environing processes resulting in distinctive, variegated and dynamic ‘individual-milieu coupling [s]’ (*ibid*.: 3); although we need to be careful not to construct an interior/exterior or individual/milieu binary, for the *milieu* is a ‘constituting energetic system… the very activity of relation, the reality of the relation between two orders that communicate across a singularity’ (*ibid*.: 50).^4^ As Brian Massumi points out, this understanding of *milieu* reflects its French meaning as ‘both “middle” and “surroundings”’:

To put the two meanings together without falling back into an outside/inside division that calls for a subject or object to found or regulate it, you have to conceive of a middle that wraps around, to self-surround, as it phases onward in the direction of the “more” of its formative openness. In a word, you have to give the precept of beginning in the middle a topological twist. (Massumi, 2013: xii)^5^

Individuated beings-in-becoming are not individuals separated from an exterior milieu/environment, but topological entanglements-in-becoming, and Simondon’s principal concepts for understanding this ‘individual-milieu coupling’ and related principles of individuation are drawn from across the natural, physical and human sciences, as well as philosophy.^6^ In part one of *Individuation* he focuses on physical individuation in non-living beings, outlining his well-known case studies of crystal growth and the moulding of clay bricks. It is here that he advances his core arguments relating to ontogenesis, individuation, matter, form and milieu through ideas drawn from physics and chemistry as well as philosophy. This includes concepts appropriated from thermodynamics – ‘the notions of potential charge, oriented tensions, supersaturation, and phase shift’ (Garelli, 2020: xx) – which help to disrupt any automatic association of processes of individuation with human individuals and collectives, while these same terms enable Simondon to approach the psychic and collective individuation of living beings in alternate ways. It is in this latter focus on ‘collective individuation’ that my primary interests lie, for Simondon is insistent that groups and collectives are ‘not a gathering of individuals’ but instead result from processes of ‘individuation’ and ‘ontogenesis’ (Simondon, 2020: 332, 333). This approach forces us to dispense with pointillist and summative approaches to being, identity, representations and places which see them as assembled or formed from heterogeneous components in a singular and linear, ‘building block’ manner. Rather, Simondon’s philosophy of transindividuality asserts that an ‘individual is multiple insofar as it is polyphasic’ (*ibid*.: 361), with the living individual being best approached as ‘agent, milieu, and element of individuation’ (*ibid*.: 236) or ‘a certain topological arrangement’ that traverses or relates ‘a milieu of interiority and a milieu of exteriority’ (*ibid*.: 250).^7^

Simondon’s concept of ‘transindividuality’ focuses attention on the transversal processes, forces and meanings which do not occur ‘between beings’ but ‘across beings’ (Simondon, 2020: 344), revealing a ‘paradoxical topology of the transindividual’ which ‘is neither interior nor exterior to the individual’ (Combes, 2013 [1999]: 41). It is, here, that scholars have drawn parallels between Simondon’s philosophy of ontogenesis, individuation and transindividuality and the writings of both Merleau-Ponty and Deleuze (see De Beistegui, 2013). Indeed, Simondon’s work exerted a direct influence on Deleuze’s writings, and in a 1966 book review of the first part of *Individuation*, Deleuze lavished much praise on Simondon’s ‘profoundly original theory of individuation’, discussing his concepts of disparation, singularity, pre-individual being, phased being and ontogenesis in great detail (Deleuze, 2001 [1966]: 43). With the publication of *Difference and Repetition* two years later, we can see how Simondon’s focus on processes of individuation shaped an important aspect of Deleuze’s non-representational philosophy, shifting his attention away from identities, representations and static, closed subjects and objects, towards ontogenetic processes relating to the ‘virtual’ and ‘actual’, and ‘differentiation’ and ‘differenciation’ (Deleuze, 2004 [1968]; Bowden, 2013).

So, what can Simondon’s thinking on individuation, ontogenesis and transindividuality contribute to geographical understandings of place? In one sense, Simondon’s writings on individuation and structuration from the 1950s might appear to resonate with debates about the structuration of spaces and places from the early 1980s (e.g. Pred, 1983, 1984, 1986; Thrift, 1983). Given the importance of Simondon’s thinking for Deleuze, it should also come as no surprise that there are strong parallels between Simondon’s thinking and Deleuzian writings on place by Doel, Thrift and others. However, Simondon’s post-human or more-than-human account of physical, biological, psychic *and* collective individuation gives rise to a philosophy of individuation which is not simply grounded in philosophy or in the ‘social’ and ‘psy’ sciences. Just as several generations of geographers and anthropologists have revealed how places are composed of and by a great many things (including built and found material forms, non-human life, and individual and collective human groupings), Simondon’s writings point to how places might be seen to emerge from multiple and multi-phased processes of individuation (and collective individuation) which have physical, social, psychic and collective aspects. Places are plurally *and* incessantly individuated from transindividual topological entanglements of differentiated living beings, physical objects and immersive environmental milieux. If we want to categorise and discipline these plural, multi-phased individualising processes, then we would find that they have social, cultural, economic, political and physical-environmental aspects to them. The problem, of course, is that such artificial ‘labels’ or sub-disciplinary categorisations themselves conform to the logics of representation and identity which Simondon and Deleuze seek to overcome.

Despite the attempts of all-manner of individuals and collective groupings to stabilise, settle and fix meanings for personal or collective political reasons, ‘progressive’ approaches to place-making and the ontogenetic becoming of places should acknowledge the dynamism and multi-phased becoming of places as individuated figurations or distillations. Places are not assembled, constructed or formed. Rather, they are best characterised as individuated distillations, deformations or perturbations that are plural, multi-phased and have political and environmental consequences. Simondon’s positing of a pre-figured ‘pre-individual’ as anterior to the becoming, unfolding or crystallisation of the individual introduces a series of seemingly abstract philosophical propositions which parallel those made by Spinoza and Deleuze in discussing affect and difference-in-itself. Indeed, Spinozan, Simondonian and Deleuzian processes of individuation cut across and interrupt Western approaches to biological and social difference built around concepts of identity and representation, and categories of social and biological stratification. For some critics, such pre-individual tendencies and affects may appear as universal, homogenised figurations, but I would echo the arguments of poststructural feminists and critical race theorists who have pointed to the ways in which the work of both Deleuze and Simondon can help geographers and others to think difference otherwise, as prior to ‘the representational practices that “construct” social categories’ (Cockayne et al., 2017: 590; Grosz, 1994; Colls, 2012; Saldanha, 2006, 2007, 2010). As Elizabeth Grosz has suggested in a series of ‘feminist reflections’ on Simondon’s writings on ‘identity and individuation’, this work can hold important lessons for feminist work on individuation, subjectification and identification, in which ‘genders, races, classes, [and] ethnicities’ are approached as ‘neither structures nor forms, neither intersected nor singular and self-identical’ but as ‘transindividual groups, that cohere not only because they share a common milieu (the environment of various forms of oppression) but also because they share some kind of internal resonance, some form of informational coding that brings together their members, in various degrees of adhesion, to social/political collectives’ (Grosz, 2013: 54).

## V Conclusions

In this paper I have focussed on geographical accounts of the processual aspects of spaces and places, spacing and placing, examining how a wide range of approaches have tried to articulate the dynamism, openness and relationality of places. Despite the popularity of engaging with relational and poststructuralist theories, I argue that few geographers have taken seriously the ramifications of non-representational, processual and non-identarian thinking for how they figure space and place. While Marcus Doel (1999) re-visions geography as *origami* – focussing on becoming, processes, folds and events – the vast majority of geographers still subscribe to pointillist ontologies incorporating relational and poststructuralist insights into constructivist and representational approaches which seek to understand localised instantiations of place-making and locally grounded constructions of place in more open, relational, processual and plural ways. Geographers clearly have important political reasons for doing this, and the ontological positions and theoretical language they utilise may be more closely aligned with dominant everyday spatial imaginaries and the language used by policy-makers. Nevertheless, here I have proposed an alternative, non-representational and processual approach to place-making which draws upon the writings of Deleuze and Simondon to consider places as individuated distillations, deformations or unfoldings, rather than as constructions. I have argued that places can usefully be conceptualised through Simondon’s understanding of crystals-in-solution, where crystals are in a process of emergence as individualised, multi-phased entities amidst an associated milieu. Such an approach can help to avoid producing singular, pointillist, ‘building block’ figurations of places and place-building *in* partially bracketed worlds or environments, as well as avoiding integrating processual and poststructuralist approaches into a qualified, constructivist empirical framework which continues to foreground representation and identity.

Another consequence of such a revised approach would be to dismantle and shift attention away from a series of distracting binaries which present an over-simplified view of the processes through which individuated subjects, objects, identities and places emerge and unfold. Deleuze’s philosophy of difference and non-representational geographies are sometimes critiqued for being relentlessly affirmative and positive, leaving little room for negation, negativity, absence, withdrawal and non-relations (Rose et al., 2021). A key problem with these critiques and debates is that they rely upon binary oppositions of positivity/negativity, affirmation/critique, addition/subtraction, assembly/disassembly and sameness/difference (see Ruez and Cockayne, 2021), presenting affirmationist thinking as focussing on worlds ‘constructed’ through addition, inclusion, presencing and building but failing miserably to account for absences, limits, attrition and voids in a way that does not render them present (Harrison, 2007; Philo, 2017; Rose et al., 2021; cf. Dekeyser, 2023). Drawing upon Deleuze and Simondon’s writings, I would argue that while these ‘negative geographies’ may raise important political questions around experience, judgement and critique, their binary language of addition and subtraction, affirmation and critique, etc. slips into a constructivist, pointillist and binarising ‘building’ approach, whereas scholars would be best placed to work across such a negative/positive binary by focussing on the incessant becoming, unfolding and transversal practices of judging, sensing and experiencing.

This rethinking of addition/subtraction and positivity/negativity brings to mind Deleuze and Guattari’s (1988 [1980]: 25) characterisation of the rhizome in *A Thousand Plateaus* as embodying ‘the conjunction, “and… and… and”’, which is not an instruction to add, add, add, but an observation that a rhizome is always becoming, in the middle of things, never a settled being. As Marcus Doel has explained, ‘whenever there is an “and,” there is *never* a clean cut separating distinct and immutable terms’, rather we must approach ‘and’ as ‘a little fold, which is itself the occasion for the intrusion of innumerable interleaved folds into our world’ (Doel, 1996: 422, 423). Deleuze’s affirmative philosophy of difference, then, traces a scrumpled, enveloped, twisting and enfolded processual geography that is not continuously ‘building, enriching, layering’ (Philo, 2017: 258), but is continuously becoming: unfolding, hollowing, individualising, decaying, settling and un/en-gathering (Doel, 1996, 1999). If these neo-vitalist Deleuzian and Simondian geographies are neither ‘expanding’ nor ‘contracting’, then are they unrealistically or over-bearingly positive in a more qualitative and experiential sense of the term, focussing only on liveliness and positive affects, feelings, forces and bodily capacities, rather than negative feelings, bodily vulnerabilities and diminished capacities (Dekeyser et al., 2022)? This question and critique has been posed by a wide range of scholars who have critically engaged with non-representational geographies and neo-vitalist writings on affect (see Simpson, 2017; Dekeyser et al., 2022), but there is nothing inherently positive about such theoretical perspectives, which can lend themselves to studies of pain, despair, hate, loss and fear as much as hope, joy and happiness (see Anderson, 2014). Indeed, Simondon’s (2020a: 3) writings on poly-phased, individuated beings-in-becoming, and the incessant transformation of ‘individual-milieu coupling[s]’ could usefully be mobilised to understand the complex entanglements of human societies with their natural, physical and environmental surroundings, perhaps focussing upon themes such as humankind’s exploitation of natural resources, their complex entanglements with non-human life, and their role in a changing climate, none of which are adequately theorised through traditional Western binaries.

## Notes

1. The English term assemblage has ‘a more restrictive range of uses’ which focus attention on the assembled object, and there is a risk that readers miss the connection with Deleuze’s thinking on ‘event, becoming, and sense’ (Phillips, 2006: 108, 109).

2. Of course, much of Deleuze and Guattari’s writings – together and separately – from the late 1960s were an attempt to move beyond the formalism of structuralist thought in semiotics, psychoanalysis and anthropology. Indeed, it is commonly stated that it was their separate critiques of structuralism which brought them together in the early 1970s (see Dosse, 2012; Smith, 2018).

3. Extracts of *On the Mode of Existence of Technical Objects* first appeared in English translation in the early 1990s at a time when Simondon’s ideas were also being rediscovered in French philosophy (LaMarre, 2013). Since then, there have been a wide range of engagements with his thinking in philosophy and cultural studies (see Stiegler, 1998 [1994], 2009 [1996], 2011 [2001]; Mackenzie, 2002; Toscano, 2006; De Boever et al., 2013; Combes, 2013 [1999]), as well as a more recent concern with his work in anglophone cultural geography (see Woodward et al., 2015; Lapworth, 2016, 2020, 2023; Roberts, 2017; Ash, 2018; Keating, 2019, 2023).

4. There are similarities, here, with Derek McCormack’s discussions of the affective forces of ‘worlding’, and of how worlds ‘are felt as a kind of circumstantial palpability’ (McCormack, 2017: 2). Perhaps ‘worlding’ could be approached as an environing process of a particular kind, reflecting distinctive ‘individual-milieu coupling[s]’ and uncouplings (Simondon, 2020 [2005]: 3).

5. *Milieu* served as an important concept for several generations of French social geographers working in the Vidalian tradition, but the double meaning Massumi discusses does not appear to have been expressed or invoked in their work (see Buttimer, 1971: 3, n.6). More recently, Engelmann and McCormack (2021: 1419, 1426) have identified *milieu* as one of four ‘orientations’ for discussing ‘elemental worlds’, and their use of the term articulates the multiple meanings and qualities of the French concept which are utilised by Simondon, Deleuze and Guattari.

6. The breadth of Simondon’s thinking is reflected in a 218-page ‘History of the Notion of the Individual’ which was prepared during his doctoral research between 1952 and 1958, and published in volume two of *Individuation in Light of Notions of Form and Information* (Simondon, 2020b [2005]).

7. Simondon is not the only social theorist or philosopher to borrow the concept of ‘phase’ and ‘phase-shift’ from physics. Three examples in geography are Arun Saldanha (2007), Martin Jones (2009) and James Ash (2018).
